# Comparison of Omega-3 Eicosapentaenoic Acid Versus Docosahexaenoic Acid-Rich Fish Oil Supplementation on Plasma Lipids and Lipoproteins in Normolipidemic Adults

**DOI:** 10.3390/nu12030749

**Published:** 2020-03-12

**Authors:** Zhi-Hong Yang, Marcelo Amar, Maureen Sampson, Amber B. Courville, Alexander V. Sorokin, Scott M. Gordon, Angel M. Aponte, Michael Stagliano, Martin P. Playford, Yi-Ping Fu, Shanna Yang, Nehal N. Mehta, Alan T. Remaley

**Affiliations:** 1 Lipoprotein Metabolism Section, Translational Vascular Medicine Branch, National Heart, Lung and Blood Institute (NHLBI), National Institutes of Health (NIH), Bethesda, MD 20892-1666, USA; alexander.sorokin2@nih.gov (A.V.S.); scott.gordon@uky.edu (S.M.G.); michael.stagliano@nih.gov (M.S.); alan.remaley@nih.gov (A.T.R.); 2 Clinical Center, Department of Laboratory Medicine, NIH, Bethesda, MD 20814, USA; msampson@cc.nih.gov; 3 Clinical Center, Nutrition Department, NIH, Bethesda, MD 20814, USA; amber.courville@nih.gov (A.B.C.); shanna.bernstein@nih.gov (S.Y.); 4 Saha Cardiovascular Research Center and Department of Physiology, University of Kentucky College of Medicine, Lexington, KY 40506, USA; 5 Proteomics Core, Cardiovascular Branch, NHLBI, NIH, Bethesda, MD 20814, USA; apontea@nhlbi.nih.gov; 6 Section of Inflammation and Cardiometabolic Diseases, Cardiovascular Branch, NHLBI, NIH, Bethesda, MD 20814, USA; playfordmp@nhlbi.nih.gov (M.P.P.); nehal.mehta@nih.gov (N.N.M.); 7 Office of Biostatistics Research, NHLBI, NIH, Bethesda, MD 20814, USA; yi-ping.fu@nih.gov

**Keywords:** eicosapentaenoic acid (EPA), docosahexaenoic acid (DHA), omega-3 fatty acids, lipoprotein metabolism, triglyceride, LDL cholesterol, HDL cholesterol

## Abstract

Background: Eicosapentaenoic acid (EPA) and docosahexaenoic acid (DHA) have both shared and different cardiovascular effects, and commonly used fish oil supplements have considerably varied EPA/DHA ratios. Aims: We compared the effects of fish oil supplements with different EPA/DHA ratios on lipoprotein metabolism. Methods: In a double-blind, randomized cross-over study, normolipidemic adults (n = 30) consumed 12 g/day of EPA-rich (EPA/DHA: 2.3) or DHA-rich (EPA/DHA: 0.3) fish oil for 8-weeks, separated by an 8-week washout period. Results: Both fish oil supplements similarly lowered plasma TG levels and TG-related NMR parameters versus baseline (*p* < 0.05). There were no changes in plasma cholesterol-related parameters due to either fish oil, although on-treatment levels for LDL particle number were slightly higher for DHA-rich oil compared with EPA-rich oil (*p* < 0.05). Both fish oil supplements similarly altered HDL subclass profile and proteome, and down regulated HDL proteins related to inflammation, with EPA-rich oil to a greater extent. Furthermore, EPA-rich oil increased apoM abundance versus DHA-rich oil (*p* < 0.05). Conclusions: Overall, fish oil supplements with varied EPA/DHA ratios had similar effects on total lipids/lipoproteins, but differences were observed in lipoprotein subfraction composition and distribution, which could impact on the use of EPA versus DHA for improving cardiovascular health.

## 1. Introduction

Fish oil supplements are one of the most commonly used non-vitamin/non-mineral natural products consumed in the United States and are used for several putative health benefits, including for their atheroprotective benefits on heart disease [1]. The main active ingredient in fish oils appears to be n-3 polyunsaturated fatty acids (PUFA), namely eicosapentaenoic acid (EPA; 20:5 n-3) and docosahexaenoic acid (DHA; 22:6 n-3). N-3 PUFA can modify a variety of cellular processes associated with lipid metabolism, inflammation, thrombosis, and atherosclerosis [2]. Results from various epidemiological and clinical studies have also demonstrated the important role of n-3 PUFA in decreasing triglycerides (TG) [3].

Although TG lowering is a consistent observation, the effect of dietary n-3 fish oil on other cardiovascular disease (CVD) risk variables are less consistent. In fact, several large clinical trials, as well as a recent meta-analysis involving more than 77,000 individuals, have failed to show a cardiovascular benefit for fish oil supplementation containing various amounts of EPA and DHA [4,5]. It is possible that the inconsistencies of fish oil studies derive from differences in their n-3 PUFA dose and study design, but also due to the differential effect of EPA and DHA on metabolic pathways related to cardiovascular disease. Regarding lipid metabolism, both complementary and divergent effects have been described for EPA and DHA. Generally, DHA, but not EPA, tends to increase plasma levels of low-density lipoprotein cholesterol (LDL-C) and high-density lipoprotein cholesterol (HDL-C) [6]. The mechanism for this difference between EPA and DHA is not fully understood, but it has been suggested to be related to their different effect on lipoprotein subfractions. The main lipoproteins, VLDL, LDL and HDL, exist in many different size subfractions, which vary in their relationship to cardiovascular risk [7,8]. Some human trials show that DHA increased LDL particle size and that EPA and DHA had different effects on HDL subfractions [9]. When purified EPA and DHA were directly compared, DHA shows a stronger effect in increasing larger HDL2 particle levels, whereas EPA decreases smaller HDL3 particle levels to a greater extent than DHA [9].

Most n-3 fish oil clinical trials have used a mixture of EPA and DHA with various ratios and have not carefully examined the potential differential impact of the two types of fatty acids (FAs) on lipids and lipoproteins [5,10]. Thus, it remains largely unknown whether fish oil supplements with different ratios of EPA to DHA may possibly differ in their cardiovascular benefit. Natural sources of fish oils can substantially vary in the relative amounts of EPA and DHA that they contain. For example, tuna oil contains more DHA than EPA, whereas anchovy and sardine oil contain more EPA than DHA [11,12]. Consequently, the molar ratio of EPA to DHA in over-the-counter fish oil supplements can vary from approximately 0.3 to 3 [13], depending on the source of fish used to extract the oil. In the current study, we used two different fish oil formulations, representing a wide range of EPA/DHA ratios (0.3 and 2.3), and compared their effect on lipid and lipoprotein levels, as well as other CVD risk factors, in healthy normolipidemic adults.

## 2. Materials and Methods

### 2.1. Fish Oil Supplement Composition

Capsules containing food-grade purified EPA-rich fish oil and DHA-rich fish oil were provided by Nippon Suisan Kaisha Ltd. (Tokyo, Japan). The FA profile of the two types of fish oil supplements shown in Table 1 were determined by gas chromatography (Japan Food Research Laboratories, Tokyo, Japan). The content of total saturated fatty acid (SAF), MUFA, and polyunsaturated fatty acid (PUFA) were comparable between the two fish oils, except that the long-chain omega-3 type is different: the ratio of EPA to DHA is 2.3 in EPA-rich fish oil, and 0.3 in DHA-rich fish oil. In addition, the taste and appearance were similar for the two types of fish oil supplements. Participants either received 12 g daily of EPA-rich oil (EPA: 3.5 g; DHA: 1.5 g) or DHA-rich oil (EPA: 1 g; DHA: 3.6 g), during each arm of the study.

### 2.2. Study Population

Forty-one adult volunteers (aged 23 to 44) were recruited at the National Institutes of Health (NIH) Clinical Center (Bethesda, MD, USA). Entry criteria required that the patients be generally healthy with no chronic or serious disease. Subjects were excluded if they were taking any lipid-lowering drugs, fish oil supplements, or if their typical fish intake was more than three servings per week. The full list of inclusion and exclusion criteria is shown in Supplemental Table S1. The study was approved by the National Heart, Lung and Blood Institute institutional review board in keeping with the Declaration of Helsinki and all subjects gave written consent (ClinicalTrial.gov registration ID: NCT02514070; FDA Investigational New Drug (IND) No.: 126882). A power analysis indicated that 30 subjects would be required to detect a difference of 10% in TG levels between baseline and fish oil treatment, assuming a *p*-value of 0.05 and power of 80%.

### 2.3. Experimental Design

**Figure 1:**
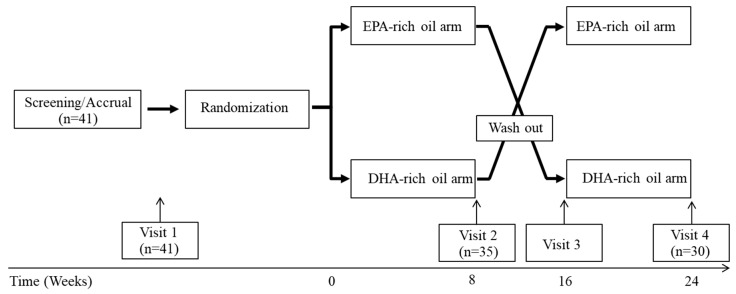
Study design. Participants were screened through an exclusion/inclusion questionnaire, baseline laboratory tests and a pregnancy test for females. Once eligibility was confirmed, subjects were randomized, and received an 8-week supply of EPA-rich or DHA-rich fish oil for 8 weeks. After an 8 weeks wash-out period, subjects received a second 8-week supply of the dietary supplement for the second arm.

The study was a randomized, cross-over, intervention study (2 × 8 weeks), with EPA-rich or DHA-rich fish oil supplement for 8-weeks, and an 8-week washout period between the two interventions (Figure 1). Subjects were instructed to take a total of 12 capsules daily (4 capsules per time, 3 times a day) during the intervention, which provided approximately 4.8 g of total EPA plus DHA. A 7-day food record and physical activity log were completed by each subject before each visit and were reviewed with nutrition staff at each visit. Subjects were counseled to maintain their lifestyle and dietary habits during the 24-week period of the study. Compliance was checked by 7-day food records, analyzed in Nutrition Data System for Research (University of Minnesota, Nutrition Coordinating Center, Minneapolis, MN, USA) and pill counting at the end of each intervention arm.

### 2.4. Biochemical Analysis

Fasting plasma lipid and lipoprotein tests that included total cholesterol (TC), high density lipoprotein cholesterol (HDL-C) and triglycerides (TG) were measured using standard enzyme coupled reactions on the Cobas 6000 analyzer (Roche Diagnostics, Indianapolis, IN, USA). Plasma low density lipoprotein cholesterol (LDL-C) was calculated by the Friedewald equation. In addition, we also used a homogenous assay (Denka Seiken Co, Ltd., Tokyo, Japan) to measure direct HDL-C, LDL-C, apoE-containing HDL (ApoE-HDL), TG-rich LDL (LDL-TG) and small dense LDL (sdLDL) as described previously [14]. ApoA-1 and apoB was measured by automated turbidometric immunoassays on the Cobas 6000 automatic analyzer (Roche Diagnostics, Indianapolis, IN, USA). Plasma levels of proprotein convertase subtilisin/kexin type 9 (PCSK9) and apolipoprotein M (apoM) were determined with ELISA kits (Abcam, Cambridge, MA, USA, and MyBioSource, Inc, San Diego, CA, USA, respectively).

To further investigate lipoprotein subclass profiles, we used an automated Vantera clinical NMR analyzer (Labcorp, Burlington, NC, USA). The LipoProfile-3 or 4 algorithm were used to measure the following lipoprotein subclass parameters: VLDL particle size (VLDL-Z) and number (VLDL-P), triglyceride-rich lipoprotein particles (TRL-P) and the following subfactions: very small-, small-, medium- and large-TRL-P; LDL particle sizes (LDL-Z) and number (LDL-P), as well as their subfractions: small-, medium-, large-LDL-P; HDL particle size (HDL-Z) and number (HDL-P), as well as their subfractions: small-HDL-P (HDL-P1~2), medium-HDL-P (HDL-P3~4), and large-HDL-P (HDL-P5~7). In addition, to investigate HDL function, plasma cholesterol efflux capacity (CEC) was estimated as described preciously using J774 cells [15]. Cholesterol efflux was calculated by using the following formula and expressed as a normalized value: ((µCi of ^3^H-cholesterol in media containing apoB-depleted subject plasma - µCi of ^3^H-cholesterol in plasma-free media)/(µCi of 3H-cholesterol in media containing apoB-depleted pooled control plasma-µCi of 3H-cholesterol in pooled control plasma-free media)). Other plasma biochemical measurements, including high sensitivity C-reactive protein (hsCRP), insulin, and glucose were performed on a Cobas 6000 analyzer (Roche Diagnostics, Indianapolis, IN, USA). The HOMA-IR index was used to estimate the degree of insulin resistance with the following formula: HOMA-IR = fasting glucose [mg/dl] * fasting insulin [mU/mL]/405.

### 2.5. Vascular Function Assessment

The heart rate (HR), blood pressure (BP), cardio-ankle vascular index (CAVI) and ankle-brachial index (ABI) were measured with the VaSera VS-1500N vascular screening system (Fukuda Denshi Co. Ltd., Tokyo, Japan) [16]. Briefly, the CAVI was estimated from the brachial and ankle pulse wave forms and electrocardiography, phonocardiography and BP measurements were simultaneously performed. ABI was measured based on the SBP for both the upper (brachial artery) and lower (tibial artery) and was calculated by dividing the ankle SBP by the brachial SBP.

### 2.6. Proteomic Analysis

Liquid chromatography–tandem mass spectrometry (LC-MS/MS) analysis was performed to investigate the HDL proteome of a random subgroup (n = 10) subjects as previously described [17]. In brief, individual plasma samples were fractionated by fast protein liquid chromatography (FPLC) system, using two Superose 6 columns in tandem on an ÄKTA Pure FPLC system (GE Healthcare Life Sciences). Eluted fractions corresponding to HDL were collected and pooled, followed by treatment with lipid removal agent (Supelco, Sigma-Aldrich (St. Louis, MO)) and trypsinization at 37 °C overnight. The resulting digested peptides were lyophilized and reconstituted in formic acid in water before analysis by LC-MS/MS on an Orbitrap Elite instrument (Thermo Scientific, Bremen, Germany). The abundance of lipoprotein-associated proteins was estimated using a semi-quantitative spectral counting method [18]. Scaffold (version Scaffold_4.1.1; Proteome Software Inc., Portland, OR) was used to validate LC-MS/MS-based peptide and protein identifications. Protein identifications were accepted if they could be established at greater than 95% probability and contained at least two identified peptides. STRING database [19] was used to perform protein-protein interactome and gene ontology (GO) enrichment analysis of altered proteins with moderate confidence (0.40).

## 3. Statistical Methods

Data are presented as the mean ± SD for parametric variables or the median ± IQR for non-parametric variables, and as absolute numbers (%) for categorical variables. Skewness and kurtosis measures were considered to assess normality and log transformations to make residuals closer to normal were employed, but because they did not affect the significance of any results, we only present untransformed data. To test the difference between fish oil supplements, students t-test for parametric variables and Mann–Whitney U test for non-parametric variables were performed. Comparisons with baseline were performed by paired t-test or Wilcoxon signed-rank test. Comparisons between EPA-rich and DHA-rich fish oils with adjustment for the period and arm effects of the baseline phase and other covariates (age, sex and racial groups) were achieved by applying a linear mixed effect model to the differences from each baseline for each biological parameter. Analysis was performed using Stata/IC 12.0 (StataCorp LP, College Station, TX, USA) and SAS 9.4 (SAS Institute Inc., Cary, NC, USA). *p* < 0.05 was considered statistically significant.

## 4. Results

### 4.1. Study Population

Baseline characteristics of the participants are shown in Table 2. The mean age was 33.9, and approximately half were male and there was a relatively wide racial distribution. Overall, the participants had a normal lipid and lipoprotein profile, with a relatively low mean TG of 89 mg/dL. Thirty out of 41 enrolled subjects completed the study. One subject withdrew because of mild alopecia (grade I) that was not attributed to the supplements. Ten subjects voluntarily withdrew because of personal time constraints related to scheduled follow-up visits as required by the protocol. There were two adverse events, including one unrelated medical condition (n = 1, during screening visit) and mild GI discomfort (grade I; n = 1, during the washout period), but both subjects completed the study. The 7-day food records showed that fish oil supplementations did not influence the mean intake of daily energy or individual nutrients, including fat, carbohydrate, protein, alcohol, cholesterol, total fiber, or individual FA content of the diet, during the study, without significant differences between the two fish oil supplements (Table 3). In addition, there were no apparent differences in terms of the tolerability of the two different fish oil supplements.

### 4.2. Characterization of Lipid/lipoprotein-Related Parameters on Supplements

Both EPA- and DHA-rich fish oil supplements similarly decreased TG by approximately 14.1% on average compared to baseline (*p* < 0.05), although there were no significant differences between EPA- and DHA-rich fish oil supplements (Table 4). There were no significant changes in TC, LDL-C, sdLDL, and LDL-TG due to either fish oil intervention, although both fish oil supplements increased apoB levels by approximately 6.1% on average compared to baseline (*p* < 0.05). No statistic differences were detected in these LDL-related parameter levels between EPA- and DHA-rich fish oil supplementations. In addition, there was no difference in PCSK9 plasma levels due to either fish oil supplement, as well as between two fish oil supplements. In terms of HDL, no changes were observed in HDL-C and apoE-HDL between baseline and either fish oil supplement, although both fish oil slightly decreased levels of apoA1 by approximately 3.5% on average compared with baseline (*p* < 0.05). There were no differences in these HDL-related parameter levels between EPA- and DHA-rich fish oil supplements. In addition, EPA-rich fish oil significantly increased apoM levels by 13.3% and 9.4% compared with baseline and DHA-rich fish oil supplement, respectively (*p* < 0.05), although there was no difference between baseline and DHA-rich fish oil supplement. Assessment of HDL function by ex vivo plasma cholesterol efflux capacity (CEC) assay did not reveal any difference in this parameter before and after either fish oil supplementation, and no significant difference was detected between the two fish oil supplements (baseline: 1.1 ± 0.2 vs. EPA-rich oil treatment: 1.1 ± 0.2 vs. DHA-rich oil treatment: 1.1 ± 0.3).

### 4.3. NMR-Determined Lipoprotein Subclass Profile on Supplements

**Figure 2:**
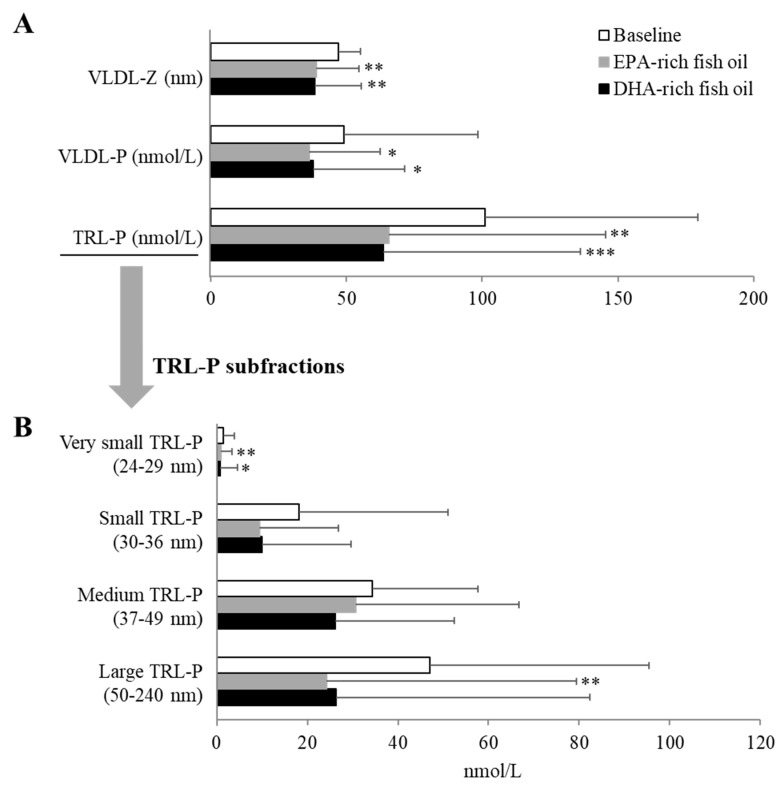
Changes in VLDL particle size/number and overall TRL-P number (**A**), and TRL-P subfraction number (**B**) after an 8-week dietary supplementation with EPA-rich or DHA-rich fish oils. VLDL-Z: VLDL particle size; VLDL-P: VLDL particle number; TRL-P: TG-Rich Lipoprotein particles. Data represented as mean ± SD (n = 30). * *p* < 0.05, ** *p* < 0.01, and *** *p* < 0.001 compared with baseline.

**Figure 3:**
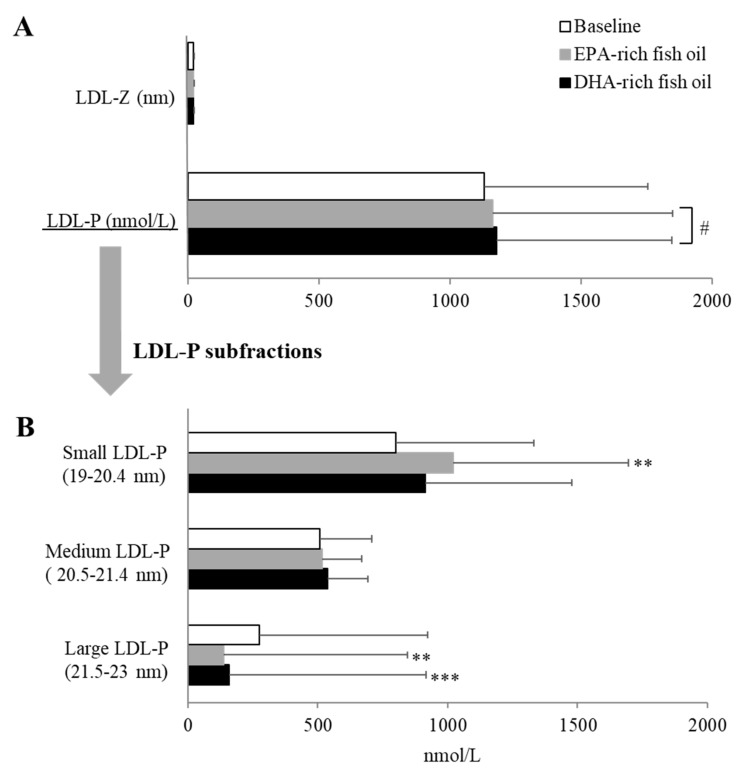
Changes in overall LDL particle size/number (**A**) and LDL subfraction particle number (**B**) after an 8-week dietary supplementation with EPA-rich or DHA-rich fish oils. LDL-Z: LDL particle size; LDL-P: LDL particle number. Data represented as mean ± SD (n = 30). ** *p* < 0.01 and *** *p* < 0.001 compared with baseline. # *p* < 0.05 between EPA-rich and DHA-rich fish oil supplements.

We further performed NMR spectroscopy to examine any possible changes in lipoprotein subclasses or particle number or size on the two different fish oil supplements. In general, the observed lipoprotein changes by routine testing were replicated by NMR, but several specific differences were observed in lipoprotein subfractions. Compared to baseline, both fish oil supplementations similarly decreased VLDL-Z, VLDL-P, and TRL-P levels by 17.9%, 24.3, and 36.2% on average, respectively (*p* < 0.05) (Figure 2A). In line with these findings, EPA- and DHA-rich fish oil showed the same trend in decreasing TRL-P subfractions (Figure 2B). Both fish oil supplements significantly reduced the numbers of very small-TRLP by approximately 41.6%, and EPA-rich fish oil significantly decreased large-TRLP levels by 40% (*p* < 0.05). For LDL subclasses, as shown in Figure 3, the overall effect of both fish oil supplements were similar, and there were no differences in measures of LDL particle size and number due to either fish oil supplementations, although DHA-rich fish oil supplement resulted in a small but significant increase (1.3%) in LDL-P levels compared with EPA-rich fish oil based on a linear mixed effect model (*p* < 0.05; Figure 3A). Large LDL-P levels were decreased by ~46% with both fish oil supplements from baseline (*p* < 0.05), and EPA-rich oil increased small LDL-P by 27.7% compared with baseline (*p* < 0.05) (Figure 3B).

**Figure 4:**
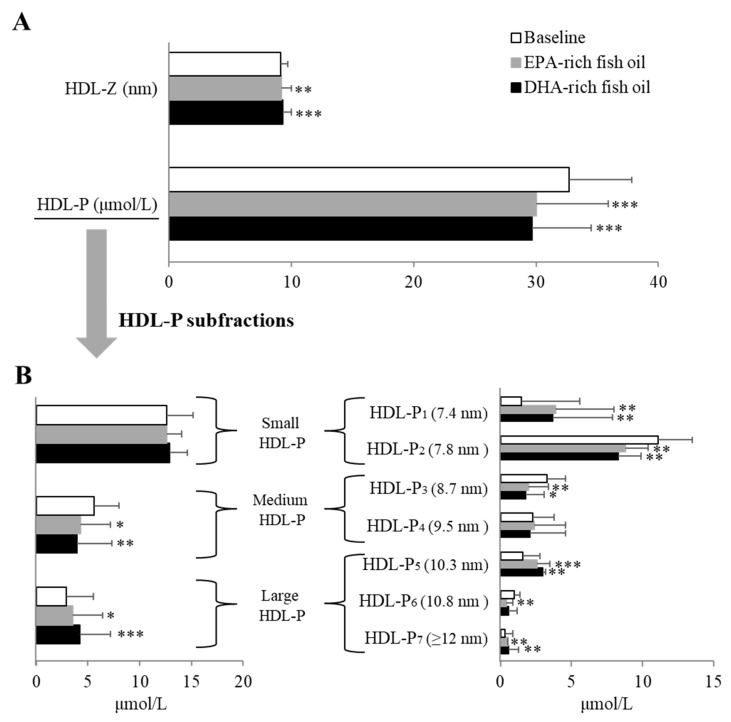
Changes in overall HDL particle size/number (**A**) and HDL particle subfraction number (**B**) after an 8-week dietary supplementation with EPA-rich or DHA-rich fish oils. HDL-Z: HDL particle size; HDL-P: HDL particle number. HDL-P1~7: HDL subspecies fraction 1~7. Data represented as mean ± SD (n = 30). * *p* < 0.05, ** *p* < 0.01, and *** *p* < 0.001 compared with baseline.

In terms of HDL, both fish oil supplements led to an increase in HDL-Z by ~1.6% on average (*p* < 0.05) and a decrease in HDL-P levels by ~9% on average (*p* < 0.05) compared with baseline (Figure 4A). Both fish oils showed similar effect on HDL-P subfractions (Figure 4B): both EPA- and DHA-rich fish oil supplements significantly increased large HDL-P (HDL-P5~7) levels by ~33% on average and decreased medium HDL-P (HDL-P3~4) levels by ~27% on average (*p* < 0.05). There was no difference in small HDL-P before or after either fish oil supplement, although both fish oils similarly changed its subfraction (HDL-P1~2): HDL-P1 levels were increased by 153% on average (*p* < 0.05) and HDL-P2 levels were decreased by 23% on average (*p* < 0.05) compared with baseline. No differences were observed in each HDL subclass parameter between EPA- and DHA-rich fish oil supplement.

### 4.4. Lipoprotein Proteome

**Figure 5:**
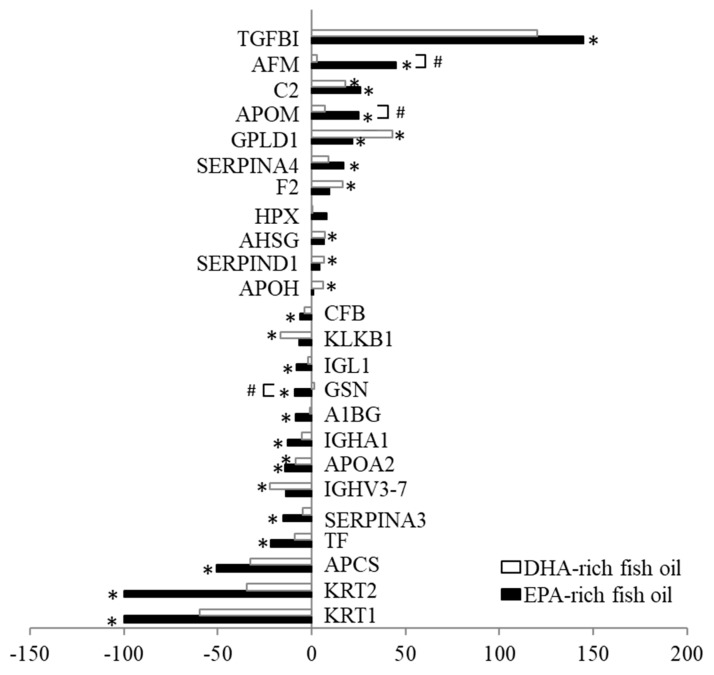
Changes (%) compared with baseline values in altered HDL-related lipoprotein proteome after an 8-week dietary supplementation with EPA-rich or DHA-rich fish oils (n = 10). TGFBI: Transforming growth factor-beta-induced protein ig-h3; AFM: Afamin; C2: Complement C2; APOM: Apolipoprotein M; GPLD1: Phosphatidylinositol-glycan-specific phospholipase D; SERPINA4: Kallistatin; F2: Prothrombin; HPX: Hemopexin; AHSG: Alpha-2-HS-glycoprotein; SERPIND1: Heparin cofactor 2; APOH: Beta-2-glycoprotein 1; CFB: Complement factor B; KLKB1: Plasma kallikrein; IGL1: Immunoglobulin lambda-1 light chain; GSN: Gelsolin; A1BG: Alpha-1B-glycoprotein; IGHA1: Immunoglobulin heavy constant alpha 1; APOA2: Apolipoprotein A-II; IGHV3-7: Immunoglobulin heavy variable 3–7; SERPINA3: Alpha-1-antitrypsin; TF: Serotransferrin; APCS: Serum amyloid P-component; KRT2: Keratin, type II cytoskeletal 2 epidermal; KRT1: Keratin, type II cytoskeletal 1. * *p* < 0.05 compared with baseline; # *p* < 0.05 between EPA-rich and DHA-rich fish oil supplements.

To examine the HDL proteome, we performed LC-MS/MS analysis of HDL fractions from 10 subjects chosen randomly from all subjects who completed the study. A total of 161 HDL-associated proteins were detected at baseline and/or either fish oil supplement, and we identified 24 proteins (11 up-regulated and 13 down-regulated proteins) that were altered with EPA- and/or DHA-rich fish oil supplementation compared to baseline (*p* < 0.05; Figure 5). Overall, the two fish oil supplements acted similarly in altering the lipoprotein proteome, but with EPA having a greater effect on the down-regulated proteome. Two out of 13 down-regulated proteins were due to DHA, while 11 were due to EPA treatment. EPA-rich fish oil also showed specific effect on several HDL-associated proteins: abundance of apolipoprotein M (APOM) and afamin (AFM) were increased by 17% and 41%, respectively, and gelsolin (GSN) was decreased by 10% with EPA-rich oil compared with DHA-rich oil supplementation (*p* < 0.05). The 24 altered HDL-related proteins were further classified according to their biological function using GO analysis. Top 10 GO terms of upregulated and downregulated proteins are shown in Table 5 and Table 6, respectively. GO analysis showed that the upregulated proteins were mainly associated with the regulation of protein metabolic processes and blood coagulation processes. The downregulated HDL-related proteins were mainly linked to the processes related to inflammation, defense and immune processes, such as acute inflammatory response, complement activation, and defense/immune response process. In addition, Supplemental Figure S1 showed the protein-protein interactome map and top 10 GO terms of APOM, AFM, and GSN that were altered by EPA-rich oil compared with DHA-rich oil supplement. The most affected GO terms of APOM were related to cholesterol efflux and lipoprotein metabolism, those of AFM were related to immune processes, and those of GSN were related to amyloid fibril formation. Plasma apoM levels were also evaluated by ELISA (Table 4) and EPA-rich fish oil supplement increased apoM by 12.9% and 9.1% compared with baseline and DHA-rich fish oil supplement, respectively (*p* < 0.05).

### 4.5. Other Risk Factors and CVD Biomarkers

Neither fish oil supplementation had a major effect on non-lipid biomarkers related to CVD (Table 7), such as hsCRP. There were also no significant changes in parameters related to glucose metabolism (fasting glucose, insulin, or HOMA-IR score). Although EPA-rich oil supplementation showed a statistically significant decrease in hemoglobin A1c (HbA1C) compared to baseline (0.14%; *p* < 0.05), the change was relatively small, within the normal range and not clinically significant. Other observed changes in clinical laboratory tests were relatively small and viewed to be clinically insignificant. In terms of vascular function parameters, there were no significant changes in ankle-brachial index (ABI), cardio-ankle vascular index (CAVI), blood pressure (BP), and heart rate before and after either fish oil intervention.

## 5. Discussion

This study addressed whether fish oil supplements with varied EPA/DHA ratios have different effects on lipoprotein metabolism in healthy normolipidemic adults. We chose two fish oil supplements from natural sources that nearly spans the range for EPA/DHA that can be found in over-the-counter fish oil supplements. Our data indicate that 12 g/day of EPA- or DHA-rich fish oil supplement (~4.8 g/d total EPA+DHA) were similarly well tolerated in normolipidemic subjects, and that both fish oil supplements similarly decreased plasma TG levels, as well as overall particle numbers of VLDL and TG-rich lipoprotein subfractions.

Although fish oils have some other clinical indications, such as for reducing the risk of acute pancreatitis from hypertriglyceridemia [20], the main driver for their use by the public has been for their perceived benefit in reducing cardiovascular disease [2]. The most recent recommendations from the American Heart Association concluded that n-3 PUFA at high doses of 4 g/d (>3 g/d total EPA+DHA) is effective in reducing TG in hyperlipidemic individuals [21]. It has not been clearly demonstrated, however, that lowering TG by other drugs like fibrates can decrease cardiovascular events [22]. The REDUCE-IT trial, which used 4 g of purified EPA in patients with modest hypertriglyceridemia, did show a substantial further reduction in cardiovascular events when used on top of statins, but it is still uncertain whether it was due to TG lowering or by some other mechanism [23]. Recently, the STRENTH trial of using 4 g of a mixture of EPA and DHA (EPA to DHA ratio: 2.75) has been halted because of a lack of an apparent effect in reducing CVD outcomes in patients with mixed dyslipidaemia [24], raising the question of the efficacy of fish oil supplements with different ratios of EPA to DHA on CVD risk. Purified EPA is only available as a prescription medication, whereas all the over-the-counter forms of fish oil supplements contain a varying mixture of EPA and DHA.

In the current study, plasma TG levels were decreased by 14.4% on average due to fish oil supplements containing a total for 4.8 g of EPA plus DHA, which agrees with previous finding showing that ≥4 g/d of n-3 PUFA results in 9%–26% reduction in circulating TG in normolipidemic to borderline hyperlipidemic individuals [3]. When comparing the effectiveness of the two fish oils used in this study, no differences in TG-lowering effect were observed, which is also in line with previous studies [25,26]. The mechanism related to TG lowering by fish oil supplementation is thought to be due to suppression of hepatic VLDL production, one of the main lipoprotein carriers of TG [27]. When we examined both VLDL-P and size and TRL-P and size by NMR, we observed that both fish oil supplements equally lowered larger particles, which are more enriched in TG.

Neither fish oil supplement in this study changed plasma levels of LDL-C as determined by the Friedewald equation or by a homogenous direct assay (Denka). Pro-atherogenic sdLDL-C also did not change, as well as plasma PCSK9, which regulates circulating LDL-C levels [28]. Both fish oil supplements, however, showed a similar small effect in raising apoB between 5.4%–6.8%, which given the greater predictive value of apoB over LDL-C on the impact of a therapy on cardiovascular outcomes suggests that neither fish oil treatment used in this study may be beneficial [29]. There was also a trend toward increased LDL-P for both fish oils consistent with changes in apoB, but it did not reach statistical significance. When LDL subfractions were individually analyzed there was a statistically significant increase in small over large LDL for both fish oil supplements, which would be expected to increase CVD risk [30]. When comparing on-treatment values, there was a small but significantly greater increase in overall LDL-P levels for DHA-rich oil compared to EPA-rich oil. Several previous studies have shown that the effect of DHA on increasing LDL-C and LDL particle size was greater than for EPA [9,10], whereas other studies showed no change in LDL-C with either EPA or DHA. It has been suggested that the effect of n-3 fish oils on LDL-C is less consistent compared with their TG-lowering effect, and depends on multiple factors, such as study period and population, as well as n-3 PUFA dose and types. The differences observed in this study in LDL-C and LDL subclass profile on the EPA vs. DHA-rich fish oil supplements need to be examined in subjects with elevated LDL-C and other types of dyslipidemias, which could reveal greater differences in these two types of fish oil supplements.

HDL, which is generally considered to be atheroprotective [31], has not been well studied in regard to the effect of fish oil supplementation on its composition and function. By meta-analysis, n-3 PUFA consumption appears to moderately increased HDL-C levels, but the effect is generally small, especially when study participants are healthy [32]. In the currently study, neither fish oil supplement resulted in changes in HDL-C or in apoE-HDL, a minor HDL subclass thought to be more cardioprotective [33]. Given the multitude of potential functions of HDL and its complicated subtraction distribution, HDL-C may be a poor metric of its function, as has been shown in the case of cholesterol efflux [31,34]. We, therefore, also examined its subfractions by NMR and observed again that the two fish oils showed nearly identical effects in causing a decrease in HDL-P. The smallest subfraction increased on both oils and this would, in general, be considered to be pro-atherogenic but another larger subfraction increased, making it difficult to predict the overall impact on CVD risk. The various proteins in HDL are also known to influence the functions of HDL [35], and the HDL proteome has been reported to respond to dietary lipid composition [36]. Another important function of HDL is its anti-inflammatory abilities that may contribute to its anti-atherogenic properties [37]. Fish oil-derived n-3 PUFAs can possibly influence inflammatory processes by a variety of mechanisms, such as shared and different roles of EPA and DHA in producing bioactive metabolites [38]. In the current study, we found the down-regulation of several proteins involved in inflammation by both fish oil supplements, but to a greater extent with EPA-rich fish oil. When compared to DHA-rich oil, EPA-rich oil significantly decreased the abundance of gelsolin, a multifunctional protein involved in multiple biological process, including modulation of inflammatory response [39]. EPA-rich oil also resulted in a greater increase in the abundance of apoM in HDL fractions compared with DHA-rich oil. This was consistent with also the greater increase in plasma levels of apoM after EPA-rich oil supplementation. ApoM has several potential anti-atherosclerotic functions [40]. ApoM forms a complex with sphingosine-1-phosphate (S1P), a potent bioactive lipid [41], and delivers it to endothelial cells where it may decrease vascular permeability. Increased S1P on HDL has been shown to be inversely related to CVD events and treatment of mice with stable analogues of S1P have been shown to reduce atherosclerosis [42]. ApoM has also been reported to enhance reverse cholesterol transport by promoting the formation of pre–β-HDL [43]. The transcription and secretion of apoM are regulated by a series of transcription factors, such as liver X receptor and forkhead box A2, that are known to be modulated by dietary nutrients, including omega-3 fish oils [44]. Previous animal studies have also showed that dietary n-3 PUFA-rich fish oil can enhance reverse cholesterol transport [45], but at least for the first step of this process (cellular cholesterol efflux) [46], we observed no difference in plasma CEC compared to baseline after either fish oil supplement. The limited and mixed findings of clinical studies on n-3 PUFAs containing different ratios of EPA and DHA warrant further investigation on the individual effect of EPA and DHA on lipoprotein subfractions, proteome, and other cardiovascular biomarkers besides those related to lipids.

### Study Strengths and Limitations

A strength of this study is its double-blind, randomized, crossover design, which maximized our ability to detect differences between the two fish oil supplements. The two fish oil supplements also had a similar taste and appearance and were well matched in their FA profile except for having different EPA/DHA ratios. In addition, no major changes were found in lifestyle and dietary factors of the participant during the course of the study. Our study does have the following limitations: the sample size was relatively small, the supplement duration was short, participants were relatively healthy although they are generally overweight, it was a single dose design, and we did not collect red blood cells to measure membrane incorporation of DHA and EPA before and after treatment. In addition, we compared a large number of variables simultaneously therefore the multiple testing problem is likely to result in some significant results simply due to chance alone. Another limitation is that more research is needed to understand the clinical relevance in regard to atherogenesis for the differences we did observe in the effect of the two supplements on the NMR lipoprotein profile and the apoM content of HDL. Furthermore, no effects on inflammation and glucose homeostasis were observed with either fish oil supplement, but further studies are still warranted to examine EPA/DHA supplementation for specific subgroups of subjects with inflammation and insulin resistance.

## 6. Conclusions

Supplementation of fish oil with approximately the highest and lowest EPA/DHA ratios that are commonly found in over-the-counter supplements showed nearly identical beneficial effects on plasma lipids and lipoprotein subclass profile in normolipidemic adults, particularly in regard to lowering plasma TG and TRL particles. Future studies are warranted to evaluate the generalizability of our findings in larger and more heterogeneous patient populations, such as those with dyslipidemias.

## Figures and Tables

**Table 1:** Fatty acid content per day of EPA-rich or DHA-rich fish oil supplement.

Major FA (g/day)	EPA-Rich Fish Oil Supplement	DHA-Rich Fish Oil Supplement
C16:0	0.89	1.46
C16:1 n-7	1.14	0.64
C18:0	0.08	0.33
C18:1 n-9	0.71	2.03
C18:2 n-6	0.13	0.15
C18:3 n-3	0.09	0.07
C20:1 n-9	0.05	0.21
C20:4 n-6	0.15	0.26
C20:5 n-3 (EPA)	3.48	0.99
C22:6 n-3 (DHA)	1.54	3.64
Total SAF	1.66	2.20
Total MUFA	2.28	3.38
EPA+DHA	5.02	4.63
EPA/DHA ratio	2.26	0.27
Total omega-3 PUFA	6.42	5.14
Total omega-6 PUFA	0.30	0.43

FA: fatty acid; SAF: saturated fatty acid; MUFA: monounsaturated fatty acid; PUFA: polyunsaturated fatty acid.

**Table 2:** Baseline characteristics of subjects.

	Measures (n = 41)
**Demographics**	
Age (years)	33.90 ± 10.67
Male sex, n (%)	17 (41.56)
Ethnicity, n (%)	
White	16 (39.02)
Black	6 (14.63)
Asian	15 (36.59)
Other	1 (2.44)
Unknown	3 (7.32)
Body mass index (kg/m^2^)	27.01 (6.43)
**Clinical and Laboratory Values**	
Systolic BP (mmHg)	120.71 ± 15.14
Diastolic BP (mmHg)	71.37 ± 10.48
TC (mg/dL)	183.78 ± 47.69
TG (mg/dL)	89.85 ± 59.49
HDL-C (mg/dL)	60.68 ± 17.79
LDL-C (mg/dL)	105.29 ± 43.81
ApoA-I (mg/dL)	150.46 ± 26.39
ApoB (mg/dL)	88.14 ± 30.39
hsCRP (mg/L)	1.77 (0.4−2.8)
HOMA-IR	2.74 (1.46-3.13)
Insulin (µU/mL)	11.81 ± 9.01
Glucose (mg/dL)	92.22 ± 10.80
HbA1C (%)	5.26 ± 0.35
AST (U/L)	19.61 ± 6.09
ALT (U/L)	19.95 ± 11.72
TSH (µlU/mL)	2.16 ± 1.17
Uric Acid (mg/dL)	5.51 ± 1.49
Creatinine (mg/dL)	0.80 ± 0.17
Albumin (g/dL)	4.42 ± 0.25
RBC (M/uL)	4.71 ± 0.48
WBC (K/uL)	5.61 ± 1.67

TC: total cholesterol; TG: triglycerides; HDL-C: HDL cholesterol; LDL-C: LDL cholesterol. Data represented as mean ± SD (n = 41) or median (IQR) for parametric and non-parametric variables respectively and as n (%) for categorical variables.

**Table 3:** Average energy and nutrient consumption without supplements based on 7-day food records before and after intervention.

Nutrient	Baseline	EPA-Rich Fish Oil	DHA-Rich Fish Oil
	(n = 30)	(n = 28)	(n = 30)
Energy (kcal)	2090.4 ± 441.1	1968.4 ± 438.7	2010.8 ± 453.1
Fat (g)	84.8 ± 24.0	78.3 ± 22.6	80.0 ± 23.4
Carbohydrate (g)	231.7 ± 72.4	222.1 ± 82.0	232.4 ± 82.7
Protein (g)	93.0 ± 23.2	89.6 ± 28.0	89.1 ± 31.1
Alcohol (g)	9.9 ± 14.4	8.1 ± 10.9	6.2 ± 7.8
Cholesterol (mg)	320.3 ± 171.0	289.9 ± 169.0	297.9 ± 118.4
Total Fiber (g)	22.9 ± 8.6	21.9 ± 7.8	23.4 ± 11.1
FA (g)			
C14:0	2.06 ± 1.07	2.00 ± 1.08	2.00 ± 1.18
C16:0	14.05 ± 4.07	12.70 ± 3.94	13.03 ± 3.65
C16:1 n-7	1.29 ± 0.56	1.13 ± 0.44	1.15 ± 0.50
C18:0	6.17 ± 2.24	5.54 ± 2.01	5.42 ± 1.80
C18:1 n-9	30.54 ± 10.11	28.23 ± 10.66	28.41 ± 10.23
C18:2 n-6	16.71 ± 5.81	15.20 ± 4.85	16.20 ± 6.30
C18:3 n-3	1.83 ± 0.89	1.74 ± 0.78	1.92 ± 1.33
C20:1 n-9	0.34 ± 0.17	0.35 ± 0.30	0.31 ± 0.19
C20:4 n-6	0.17 ± 0.09	0.16 ± 0.09	0.17 ± 0.09
C20:5 n-3 (EPA)	0.04 ± 0.06	0.07 ± 0.08	0.06 ± 0.09
C22:1 n-9	0.05 ± 0.08	0.06 ± 0.09	0.06 ± 0.12
C22:5 n-3	0.03 ± 0.03	0.03 ± 0.04	0.04 ± 0.06
C22:6 n-3 (DHA)	0.09 ± 0.12	0.14 ± 0.17	0.16 ± 0.22
Total SFA	25.9 ± 8.4	23.7 ± 7.9	24.0 ± 7.9
Total MUFA	32.5 ± 10.6	30.2 ± 11.3	30.3 ± 10.8
Total PUFA	19.0 ± 6.3	17.5 ± 5.3	18.7 ± 7.3
Total *trans* FA	1.9 ± 1.1	1.7 ± 1.1	1.7 ± 1.1
Total omega-3 PUFA	2.0 ± 0.9	2.0 ± 0.8	2.2 ± 1.3
Total omega-6 PUFA	17.1 ± 5.9	15.5 ± 4.9	16.5 ± 6.3

FA: fatty acid; SFA: saturated fatty acid; MUFA: monounsaturated fatty acid; PUFA: polyunsaturated fatty acid. Data were based on 7-day food records without supplement before each visit and represented as mean ± SD.

**Table 4:** Lipoprotein biomarker values before (baseline) and after an 8-week ingestion of EPA-rich or DHA-rich fish oil supplement.

	Baseline (n = 30)	EPA-Rich Fish Oil (n = 30)	DHA-Rich Fish Oil (n = 30)	*p*-Value (EPA-Rich Oil vs. DHA-Rich Oil)
**Lipid/Lipoprotein Profile**				
TG (mg/dL)	95.3 ± 66.6	81.7 ± 64.2 *	82.1 ± 57.9 *	0.43
TC (mg/dL)	190.9 ± 52.7	191.1 ± 49.5	193.5 ± 54.4	0.68
LDL-C (mg/dL)	110.1 ± 49.6	111.4 ± 49.8	113.8 ± 52.5	0.12
Direct LDL-C (mg/dL)	115.7 ± 48.6	114.8 ± 51.3	120.6 ± 49.6	0.95
sdLDL (mg/dL)	36.1 ± 19.9	37.3 ± 22.9	39.1 ± 23.6	0.42
LDL-TG (mg/dL)	13.1 ± 4	12.9 ± 4.3	12.8 ± 4	0.39
HDL-C (mg/dL)	62 ± 18.9	63.4 ± 20.2	63.4 ± 20.4	0.37
Direct HDL-C (mg/dL)	62.4 ± 16.8	60 ± 14.8	63.9 ± 17.7	0.24
ApoE-HDL (mg/dL)	5.6 ± 1.8	5.4 ± 1.6	5.9 ± 1.9	0.73
**Apolipoproteins**				
ApoA1 (mg/dL)	152.3 ± 27.6	147.1 ± 27.4 *	146.7 ± 26.2 **	0.8
ApoB (mg/dL)	91.8 ± 34.1	98 ± 34.4 *	96.8 ± 35.4 *	0.38
ApoM (ng/mL)	2.26 ± 0.1	2.56 ± 0.41 **^#^	2.34 ± 0.2	0.02
PCSK9 (ng/mL)	13.30 ± 3.62	14.11 ± 4.36	12.44 ± 3.48	0.64

TG: triglyceride; TC: total cholesterol; LDL-C: LDL cholesterol; sdLDL: small dense LDL; VLDLp: oxLDL: oxidized LDL; LDL-TG: TG concentration in LDL; HDL-C: HDL cholesterol; ApoE-HDL: ApoE-containing HDL; PCSK9: proprotein convertase subtilisin/kexin type 9; CEC: cholesterol efflux capacity. Data represented as mean ± SD (n = 30). * *p* < 0.05, ** *p* < 0.01. Compared with baseline. ^#^ *p* < 0.05 compared with DHA-rich fish oil supplementation.

**Table 5:** Top 10 enriched Gene Ontology (GO) terms of up-regulated HDL proteins due to fish oil supplements.

GO term	FDR	Protein ID									
		TGFB1	C2	APOM	GPLD1	SERPINA4	F2	HPX	AHSG	SERPIND1	APOH
Regulation of proteolysis	0.00087		●		●	●	●		●	●	
Regulation of humoral immune response	0.0029		●				●	●			
Protein activation cascade	0.0029		●				●				●
Negative regulation of fibrinolysis	0.0034						●				●
Platelet degranulation	0.0045					●			●		●
Blood coagulation, intrinsic pathway	0.0045						●				●
Protein metabolic process	0.0045	●	●	●	●		●	●	●		●
Negative regulation of proteolysis	0.0045				●	●	●		●	●	
Regulation of protein metabolic process	0.0091	●	●		●	●	●	●	●	●	
Chondrocyte differentiation	0.0135									●	

FDR: false discovery rate; TGFBI: transforming growth factor-beta-induced protein ig-h3, C2: complement C2; APOM: apolipoprotein M; GPLD1: phosphatidylinositol-glycan-specific phospholipase D; SERPINA4: kallistatin; F2: prothrombin; HPX: hemopexin; AHSG: alpha-2-HS-glycoprotein; SERPIND1: heparin cofactor 2; APOH: andbeta-2-glycoprotein 1. Data were from random 10 patients. The pathway analysis of the altered proteins was performed using STRING database [19] with moderate confidence (0.40).

**Table 6:** Top 10 enriched Gene Ontology (GO) terms of down-regulated HDL -proteins due to fish oil supplements.

GO term	FDR	Protein ID									
		KRT1	APCS	TF	SERPINA3	APOA2	A1BG	GSN	IGL1	KLKB1	CFB
Acute inflammatory response	2.56E-05	●	●		●	●				●	
Defense response	2.56E-05		●		●	●		●	●	●	●
Protein activation cascade	2.56E-05	●							●	●	●
Complement activation	0.00051	●							●		●
Immune effector process	0.00062	●			●		●	●	●		●
Immune response	0.00062	●	●		●		●	●	●		●
Negative regulation of wound healing	0.00069	●	●							●	
Leukocyte mediated immunity	0.0011	●			●		●	●	●		
Regulation of acute inflammatory response	0.0013		●							●	●
Regulated exocytosis	0.0013	●		●	●		●	●	●		

FDR: false discovery rate; KRT1: Keratin, type II cytoskeletal 1; APCS: serum amyloid P-component; TF: serotransferrin; SERPINA3: alpha-1-antitrypsin; APOA2: apolipoprotein A-II; A1BG: alpha-1B-glycoprotein; GSN: gelsolin; IGL1: immunoglobulin lambda-1 light chain; KLKB1: plasma kallikrein; CFB: complement factor B. Data were from random 10 patients. The pathway analysis of the altered proteins was performed using STRING database [19] with moderate confidence (0.40).

**Table 7:** Various biomarkers for CVD before (baseline) and after a 8-week ingestion of EPA-rich or DHA-rich fish oil supplement.

	Baseline (n = 30)	EPA-Rich Fish Oil (n = 30)	DHA-Rich Fish Oil (n = 30)
hsCRP (mg/L)	1.77 × (0.4–2.8)	1.76 × (0.3–2.3)	1.48 × (0.4–1.6)
(0–4.99)			
PCSK9 (ng/mL)	13.3 ± 3.6	14.1 ± 4.4	12.4 ± 3.5
AST (U/L)	19.00 ± 4.93	20.93 ± 4.76*	20.00 ± 5.11
(0–32)			
ALT (U/L)	19.57 ± 9.63	22.82 ± 12.01	20.74 ± 9.34
(0–33)			
TSH (µlU/mL)	2.09 ± 0.98	2.06 ± 0.77	2.27 ± 1.17
(0.27–4.20)			
Uric Acid (mg/dL)	5.67 ± 1.54	5.67 ± 1.49	5.60 ± 1.64
(2.4–5.8)			
Creatinine (mg/dL)	0.79 ± 0.17	0.79 ± 0.14	0.78 ± 0.15 *
(0.51–0.95)			
RBC (M/uL)	4.71 ± 0.49	4.72 ± 0.44	4.64 ± 0.43 *
(3.93–5.22)			
WBC (K/uL)	5.50 ± 1.45	5.22 ± 1.42	5.37 ± 1.65
(3.98–10.04)			
Glucose (mg/dL)	94.23 ± 11.48	96.22 ± 12.21	97.74 ± 15.01
(74–99)			
HbA1C (%)	5.29 ± 0.37	5.15 ± 0.51 **	5.29 ± 0.42
(4–6)			
Insulin (µU/mL)	11.74 ± 9.42	12.63 ± 10.04	14.93 ± 19.68
(2.6–24.9)			
HOMA IR	2.74 (1.46−3.13)	3.11 (1.48−3.64)	3.92 (1.42−3.34)
**Vascular Parameters**			
L-ABI	1.05 ± 0.09	1.04 ± 0.08	1.05 ± 0.09
R-ABI	1.03 ± 0.09	1.04 ± 0.09	1.05 ± 0.08
L-CAVI	6.2 2 ± 0.94	6.16 ± 1.02	6.22 ± 0.88
R-CAVI	6.16 ± 0.98	6.16 ± 1.04	6.22 ± 0.93
Systolic BP (mmHg)	116.96 ± 13.29	114.28 ± 10.59	112.72 ± 11.80
(120–140)			
Diastolic BP (mmHg)	68.25 ± 10.71	67.96 ± 8.24	67.32 ± 8.29
(80–90)			
Heart rate (bpm)	66.65 ± 10.77	67.16 ± 10.49	65.8 ± 12.27

hsCRP: high-sensitivity C-reactive protein; AST: aspartate aminotransferase; ALT: alanine aminotransferase; TSH: thyroid-stimulating hormone; RBC: red blood cells; WBC: white blood cells; HbA1C: hemoglobin A1c; HOMA-IR: homeostatic model assessment of insulin resistance; L-ABI: left ankle-brachial index; R-ABI: right ankle-brachial index; L-CAVI: left cardio-ankle vascular index; R-CAVI: right cardio-ankle vascular index; BP: blood pressure. Brackets indicate clinical reference ranges. Data represented as mean ± SD (n = 30) or median (IQR) for parametric and non-parametric variables respectively. * *p* < 0.05, ** *p* < 0.01 compared with baseline.

## Supplementary Materials

The following are available online at https://www.mdpi.com/2072-6643/12/3/749/s1, Table S1: The list of inclusion and exclusion criteria; Figure S1: Protein-protein interactome map and top 10 enriched GO terms of HDL-related APOM (A), AFM (B), and GSN (C) significantly altered by EPA-rich fish oil compared with DHA-rich fish oil supplementation based on STRING database (http://string-db.org/) with moderate confidence (0.40). The HDL fractions were isolated by fast protein liquid chromatography (FPLC) from a subgroup of random 10 subjects. GO: gene ontology; FDR: false discovery rate.

## Abbreviations

CVD	cardiovascular disease
DHA	docosahexaenoic acid
EPA	eicosapentaenoic acid
FA	fatty acid
TC	total cholesterol
TG	triglyceride
VLDL	very-low-density lipoprotein
LDL-C	low-density lipoprotein cholesterol
HDL	high-density lipoprotein cholesterol
TRLP	TG-rich lipoproteins (TRLP)
