# Supplementary material for: Comparison of Omega-3 Eicosapentaenoic Acid Versus Docosahexaenoic Acid-Rich Fish Oil Supplementation on Plasma Lipids and Lipoproteins in Normolipidemic Adults
Source: Nutrients. 2020 Mar 12;12(3):749. doi: 10.3390/nu12030749 (PMC7146314; doi:10.3390/nu12030749)
Supplement: Supplementary file 1 [file nutrients-12-00749-s001.pdf]

Supplemental Materials

Supplemental Table 1 (Table S1). the list of inclusion and exclusion criteria

Recruitment criteria	
Inclusion	• Male and female participants 18 years of age or above.<br>• Subject must be healthy, with no known history of cardiovascular disease.<br>• Pre-menopausal or women of childbearing potential must be non-lactating and using an effective form of birth control during the course of the study.<br>• Subject understands protocol and provides written, informed consent in addition to a willingness to comply with specified follow-up evaluations.
Exclusion	• Pregnancy, planned pregnancy (within the study period) or women currently breastfeeding.<br>• Subjects with weight changes greater than 20% over the past 3 months.<br>• Subjects planning a significant change in diet or exercise levels.<br>• Subjects already consuming more than 1.5 g per day of EPA/DHA in any form.<br>• Known sensitivity or allergy to fish, shellfish or omega-3 fatty acids supplements<br>• Subjects with known bleeding disorders (for example, Hemophilia)<br>• Subjects previously diagnosed with atrial fibrillation<br>• Subjects with clinically diagnosed hepatic disease (including but not limited to auto immune disease, hepatitis and cirrhosis)<br>• Subjects with chronic diarrhea, gastric bypass or lap-band procedures, ostomies, bowel motility problems, or other conditions that could affect intestinal fat absorption<br>• Subjects with any acute and life-threatening condition, such as prior sudden cardiac arrest, acute myocardial infarction (last six months), stroke, embolism<br>• Liver enzymes (AST or ALT) levels above 3x upper limit of normal<br>• Subjects with a TSH greater than 1.5xULN or clinical evidence of hypo or hyperthyroidism<br>• Subjects taking supplements or medications that affect lipoproteins for at least the past 8 weeks, such as fish oil supplements, bile-acid sequestrants, plant sterol supplements, fibrates, statins or Niacin.<br>• Subjects with hemoglobin <10 g/dL<br>• Subject with platelet counts <60x103/microliter<br>• Subjects with uncontrolled hypertension (resting blood pressure > 160 mmHg systolic and /or > 100 mm Hg diastolic)<br>• Subject with uncontrolled diabetes (HbA1c ≥10)<br>• Subjects who consume excessive alcohol<br>• Subject participating in other clinical studies and/or receiving other investigational drug products prior to randomization<br>• Subject taking PCSK9 inhibitors within 8 weeks prior to enrollment<br>• Subjects being treated with tamoxifen, estrogens, or progestins that have not been stable for >4 weeks.

	• Subjects initiating new medications or patients on multiple medications may also be excluded according to investigator discretion<br>• Anticipated surgery during the study period<br>• Blood donation in the last 2 weeks or planned blood donation during the study<br>• Subjects requiring regular transfusions for any reason<br>• Subjects may also be excluded for any reason that may compromise their safety or the accuracy of research data.

Supplemental Fig. 1 (Figure S1)

Protein-protein interactome map and top 10 enriched GO terms of HDL-related APOM (A), AFM (B), and GSN (C) significantly altered by EPA-rich fish oil compared with DHA-rich fish oil supplementation based on STRING database (http://string-db.org/) with moderate confidence (0.40). The HDL fractions were isolated by fast protein liquid chromatography (FPLC) from a subgroup of random 10 subjects. GO: gene ontology; FDR: false discovery rate.

A

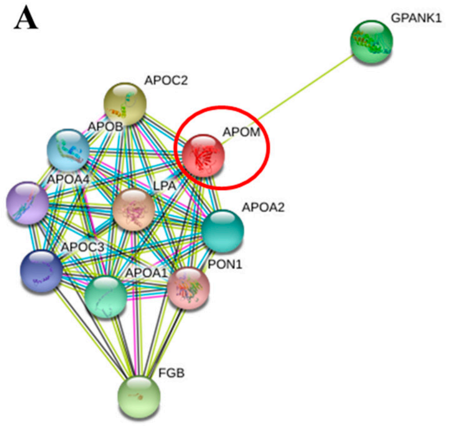

GO ID	(FDR)	GO term
0034369	(5.52e-18)	plasma lipoprotein particle remodeling
0033344	(5.51e-16)	cholesterol efflux
0034377	(5.51e-16)	plasma lipoprotein particle assembly
0034371	(1.35e-15)	chylomicron remodeling
0034378	(3.27e-15)	chylomicron assembly
0043691	(3.49e-14)	reverse cholesterol transport
0034375	(3.49e-14)	high-density lipoprotein particle remodeling
0042632	(1.77e-13)	cholesterol homeostasis
0043062	(3.42e-13)	extracellular structure organization
0001523	(5.80e-13)	retinoid metabolic process

B

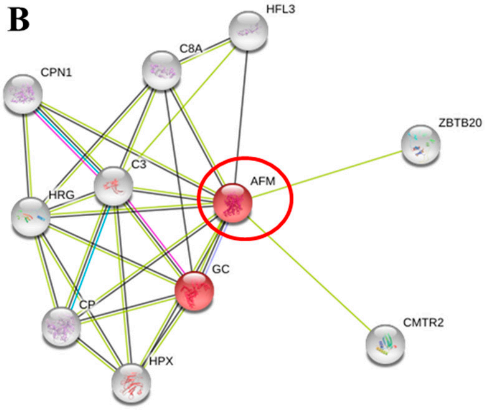

GO ID	(FDR)	GO term
0002920	(1.84e-07)	regulation of humoral immune response
2000257	(5.49e-06)	regulation of protein activation cascade
0030449	(5.49e-06)	regulation of complement activation
0002673	(2.46e-05)	regulation of acute inflammatory response
0070613	(4.83e-05)	regulation of protein processing
0002697	(7.63e-05)	regulation of immune effector process
0050776	(0.00022)	regulation of immune response
0015886	(0.00098)	heme transport
0031347	(0.0011)	regulation of defense response
0080134	(0.0014)	regulation of response to stress

C

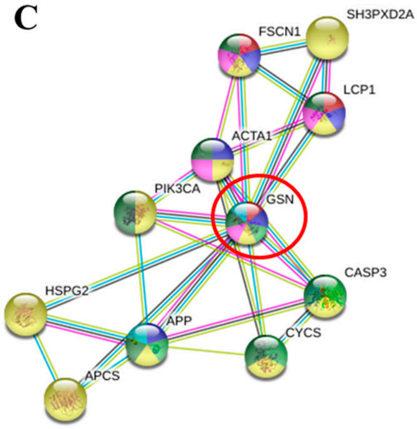

GO ID	(FDR)	GO term
0071801	(0.00014)	regulation of podosome assembly
0097435	(0.00029)	supramolecular fiber organization
0043280	(0.00029)	positive regulation of cysteine-type endopeptidase activity involved in apoptotic process
0016043	(0.00029)	cellular component organization
0007015	(0.00052)	actin filament organization
0070887	(0.0015)	cellular response to chemical stimulus
1990000	(0.0016)	amyloid fibril formation
1905906	(0.0016)	regulation of amyloid fibril formation
0030198	(0.0016)	extracellular matrix organization
0071803	(0.0018)	positive regulation of podosome assembly
